# Dynamic learning of individual-level suicidal ideation trajectories to enhance mental health care

**DOI:** 10.1038/s44184-024-00071-0

**Published:** 2024-06-07

**Authors:** Mathew Varidel, Ian B. Hickie, Ante Prodan, Adam Skinner, Roman Marchant, Sally Cripps, Rafael Oliveria, Min K. Chong, Elizabeth Scott, Jan Scott, Frank Iorfino

**Affiliations:** 1 Brain and Mind Centre, The University of Sydney, Sydney, NSW Australia; 2 Translational Health Research Institute, Western Sydney University, Sydney, NSW Australia; 3 School of Computer, Data and Mathematical Sciences, Western Sydney University, Sydney, NSW Australia; 4 Human Technology Institute, University of Technology, Sydney, NSW Australia; 5 School of Mathematical and Physical Sciences, University of Technology Sydney, Sydney, NSW Australia; 6 Data61, CSIRO, Sydney, NSW Australia; 7 Academic Psychiatry, Institute of Neuroscience, Newcastle University, Newcastle, UK

**Keywords:** Health care, Risk factors

## Abstract

There has recently been an increase in ongoing patient-report routine outcome monitoring for individuals within clinical care, which has corresponded to increased longitudinal information about an individual. However, many models that are aimed at clinical practice have difficulty fully incorporating this information. This is in part due to the difficulty in dealing with the irregularly time-spaced observations that are common in clinical data. Consequently, we built individual-level continuous-time trajectory models of suicidal ideation for a clinical population (*N* = 585) with data collected via a digital platform. We demonstrate how such models predict an individual’s level and variability of future suicide ideation, with implications for the frequency that individuals may need to be observed. These individual-level predictions provide a more personalised understanding than other predictive methods and have implications for enhanced measurement-based care.

## Introduction

Routine outcome monitoring has been promoted to enhance mental health care. The aim is to record the longitudinal characteristics of an individual’s mental health using digital tools, such as websites and apps with patient-report outcome measures^1–6^, and passive monitoring or wearable devices^7,8^.

Within the clinical setting, this routine assessment, typically termed measurement-based care (MBC^9–13^), provides more objective insights into a person’s condition, treatment response and progress. This approach improves clinical decision-making about treatment adjustments, ensuring that interventions are tailored to a person’s needs^14^. It has been shown to improve outcomes, retention, monitoring and clinician-individual communication^15–17^. As such, MBC has often been recommended for use in clinical services, and several digital technologies have been developed to help implement it (e.g. Innowell^3^, NovoPsych and Greenspace).

While MBC enhances the quality of care, interpreting measurements within an individual’s context for personalised decisions is crucial. Clinicians require predictive capability as interventions are made with the goal of improving an individual’s future mental health and functioning^18^. Unfortunately, the predictive performance of clinicians tends to be poor^19–21^, emphasising the need for tools to improve this capability.

The use of predictive models can provide insight into an individual’s likelihood of a specific outcome, although their compatibility with the ongoing and personalised nature of MBC is less clear^22,23^. Typically, they predict outcomes over defined periods, and they may not update dynamically with new observations. An alternative approach is to analyse and predict trajectories of outcomes over time, offering insight into an individual’s progress for ongoing decision-making^24–26^. However, current implementations are limited as they make predictions from a baseline or current observation and typically fail to incorporate historical observations, which hinders their practical use in real-world settings.

Categorical predictions for suicidal thoughts and behaviours are challenging, with models only performing slightly better than chance with respect to positive predictive value^27–29^. While a number of factors have been shown to be associated with suicidal thoughts and behaviours including; prior suicidal thoughts and behaviours, clinical diagnoses and specific symptoms^28,30^, functioning^31,32^ and socio-demographic factors^33^, this has not translated to high positive predictive values. To improve our understanding and prediction there is increased focus on higher frequency (e.g. hours, days or weeks) longitudinal observations. These studies have found that suicidal thoughts fluctuate over short time intervals with differences across groups of individuals^34,35^, and due to a range of factors^36^ including negative mood, hopelessness, loneliness, affective instability and poor sleep^37,38^. Recognising this fluctuation and understanding the range of possible trajectories for an individual is crucial given the inherent challenges of prediction.

Considering these issues, we advocate for a continuous time modelling framework to supplement MBC^39^. Continuous time models have several properties that make them appealing for clinically collected data including the ability to; (1) deal with irregularly time-spaced intervals and missing domains^40,41^; (2) be framed in a hierarchical manner such that parameters can be learned for the individual but constrained by the population-level^42,43^; (3) be framed in a Bayesian framework to understand uncertainties and thus the range of possible outcomes, and (4) have their underlying state values transformed onto the typically bounded and discretised (e.g. Likert) scales via link functions. Continuous time models have been applied in many fields (e.g. finance for 50 years^44^), yet their application in mental health is limited with discrete time models as the norm for frequent data sampling methods. We suspect that the properties of continuous time models make them particularly appropriate for modelling naturalistically collected clinical data within an MBC framework, which to our knowledge, has not yet been explored.

In this paper, we explore the use of continuous time models for naturally collected data in a clinical setting. We focus solely on building and comparing several model parameterisations for longitudinal data on suicidal ideation. After selecting the most appropriate model, we then show specific insights that can be learned from these models that could aid clinical decision-making.

## Results

### Sample characteristics

The sample consisted of 585 people, 72.6% female, with a mean age of 24.2 years (SD, 10.8 years). Of those included in the analysis, 238 (40.7%) had prior suicidal plans or attempts within their lifetime and 61 (10.4%) reported plans or attempts within the last three months. The mean number of observations for suicidal ideation per individual was 4.5, although it was skewed to smaller numbers with a median of 2, and more specifically 55 people with at least 10 and 140 people with at least 5 observations. The time difference between observations had a median of 35 days (IQR, 1–108 days). Other baseline characteristics of the sample are shown in Table 1.

**Table 1:** Baseline characteristics of the study participants used for modelling suicidal ideation

	Total	Female	Male
No. (%)	585	425 (72.6%)	160 (27.4%)
Demographics			
Age, mean (SD)	24.2 (10.8)	23.4 (9.9)	26.6 (12.8)
Urban^a^, No. (%)	503 (86.0%)	364 (85.6%)	139 (86.9%)
Health history, No. (%)			
Any prior mental health problem	399 (68.2%)	289 (68.0%)	110 (68.8%)
Any prior mental health treatment	402 (68.7%)	287 (67.5%)	115 (71.9%)
Any traumatic event	221 (37.8%)	164 (38.6%)	57 (35.6%)
Any physical health problem	175 (29.9%)	132 (31.1%)	43 (26.9%)
Any family mental health problem	316 (54.0%)	235 (55.3%)	81 (50.6%)
Prior suicidal plans or attempts (CSSRS, Question 3)	238 (40.7%)	169 (39.8%)	43 (26.9%)
Three-month prior suicidal plans or attempts (CSSRS, Question 4)	61 (10.4%)	47 (11.1%)	14 (8.8%)
Mental health, median (Q1, Q3)			
Distress (K10)	31 (25, 36)	32 (26, 37)	30 (23, 36)
Depressed mood (QIDS)	14 (10, 18)	15 (11, 18)	13 (9, 16)
Anxiety (OASIS)	10 (6, 13)	10 (6, 13)	10 (6, 12)
Psychosis-like experiences (PQ16)	4 (1, 7)	4 (1, 7)	4 (2, 7)
Manic-like experiences (ASRM)	2 (0, 4)	2 (0, 4)	3 (1, 5)
Suicidal ideation (SIDAS)	5 (0, 17)	5 (0, 17)	5 (0, 14)
Social support (Schuster’s SSS)	7 (5, 9)	7 (4, 9)	6 (5, 8)

^a^Locations could not be determined for 39 individuals.

### Model comparison

The Wiener process (i.e. continuous time random walk process) with random effects for all parameters was the best predictive model (see Table 2). Widely applicable information criteria (WAIC^45–47^) estimates were calculated as a measure of model performance using a subset of the data for individuals with ≥10 observations (*N* = 55). Various model formulations were explored by allowing random effects for different parameters. Initially, we assumed a Wiener process with mixed effects (random baseline but fixed diffusion). The Wiener process with random effects performed much better, which suggested that the magnitude of diffusion is different across individuals. The Ornstein–Uhlenbeck process (i.e. continuous time autoregressive process) adds an extra effect that drives individuals towards a constant value over time. We use the Wiener process models with random effects for all further analyses in this paper.

**Table 2:** Comparison of predictive performance

		Effects				
Number	Type	Baseline (${\phi }_{.,1}$)	Diffusion $({\phi }_{.,2})$	Drift (${\phi }_{.,3}$)	Constant (${\phi }_{.,4}$)	WAIC
4	Ornstein–Uhlenbeck	Random	Random	Random	Random	5390.4
3		Random	Random	Fixed	Random	5453.4
2	Wiener	Random	Random	NA	NA	5303.8
1		Random	Fixed	NA	NA	5460.2

The first two columns provide a model descriptor with a number that increases with the number of free parameters and model type. The next four columns outline whether the parameters were random or fixed. The parameters define the initial state (${\phi }_{.1}$), diffusion (${\phi }_{.2}$), lagged autoregressive effect (${\phi }_{.3}$) and a long-term constant (${\phi }_{.4}$). Further detail regarding these parameters can be found in Supplementary Methods 2. The final column provides a measure of model performance estimated using the WAIC.

### Population and individualised parameter distributions

**Fig. 1:**
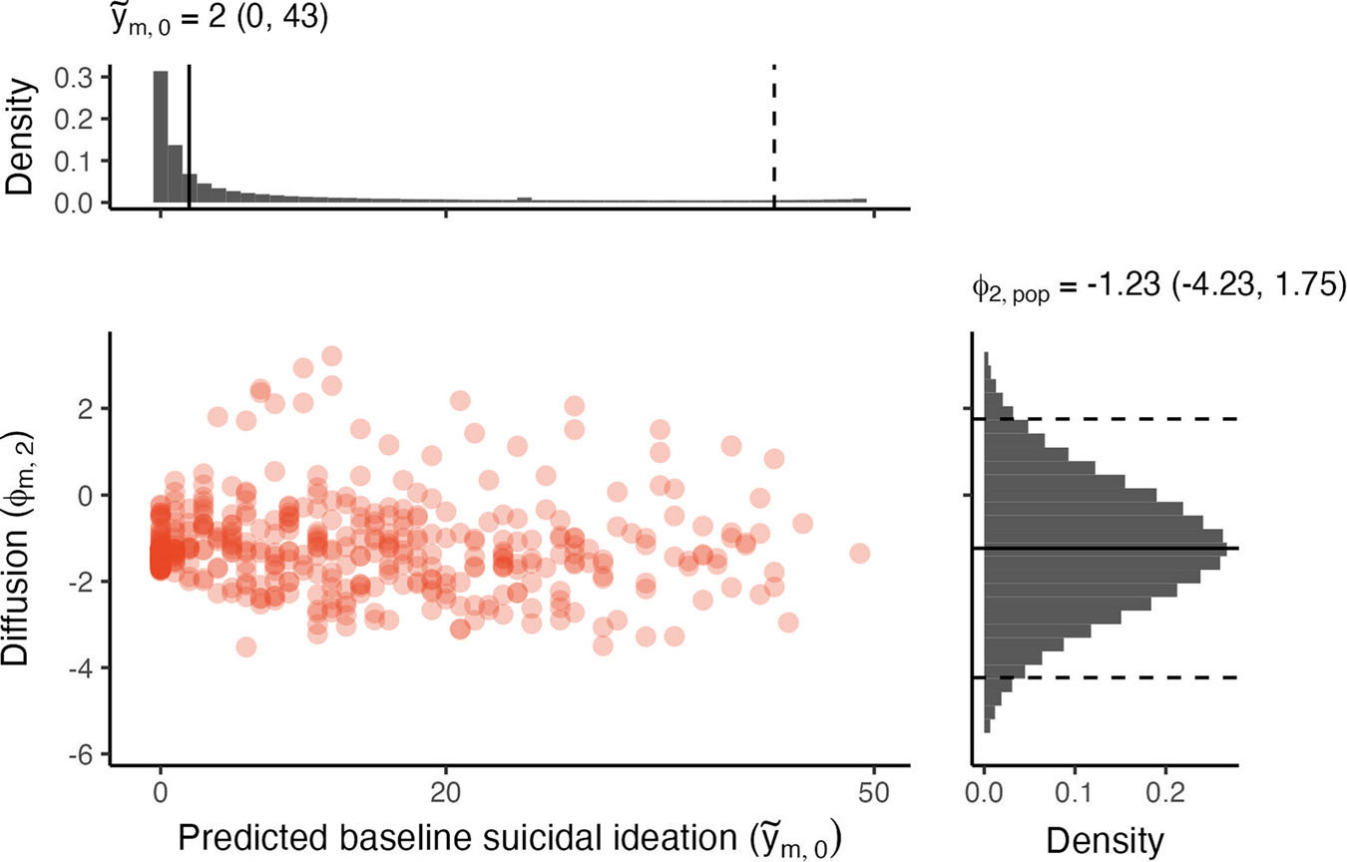
The joint distribution for the median values of the individual-level parameters. We show the median predicted baseline observation (${\widetilde{y}}_{m,0}$) compared to the diffusion parameter (${\phi }_{m,2}$) for each individual $m$. This is compared to the prior predictive distribution for each parameter given the population-level parameters, where we show the median and 95% highest density credible intervals (HDI^66^).

Our final model has two key parameters that represent a transformed baseline observation (${\phi }_{m,1}$) and a diffusion over time parameter (${\phi }_{m,2}$) for each individual $m$. We estimated a full posterior distribution for both variables for each individual. In Fig. 1 we show the median of the diffusion parameter and the predicted baseline observation, which is a transformation of the baseline parameter to the suicide ideation scale. The marginal histograms show the range of values that an individual could have given the sampled population parameters (see Supplementary Methods 5 for further detail). We see no clear dependency between these two parameters. Although, there is a large group of individuals that have zero suicidal ideation at baseline.

The diffusion is an unbounded model parameter where higher values correspond to greater observed variability in the trajectory. The values are somewhat abstract given their non-linear mapping to the observational space, but as an example, assuming a starting suicidal ideation score of 2 and the median population diffusion parameter of −1.23 a trajectory will diffuse to a range of [1, 4] within a week.

We also check for correlations between the diffusion parameter and the sample characteristics at baseline (see Table 3). Here, we find that the diffusion parameter is positively correlated with the last three-month history of suicide attempts or planning (mean, 0.10, ETI [95% equal-tailed interval], 0.02, 0.19) and borderline correlated with mania-like experiences (mean, 0.07, ETI, 0.00, 0.14).

**Table 3:** Correlation mean (95% ETI) between baseline characteristics and the diffusion parameter

Demographics	Correlation
Age	−0.01 (−0.08, 0.07)
Sex (female–male)	−0.19 (−0.53, 0.15)
Urban (urban–regional)	−0.17 (−0.40, 0.05)
Health history	
Any prior mental health problem	0.01 (−0.06, 0.08)
Any prior mental health treatment	−0.01 (−0.08, 0.07)
Any traumatic event	0.02 (−0.06, 0.09)
Any physical health problem	−0.01 (−0.08, 0.07)
Any family mental health problem	0.00 (−0.08, 0.07)
Prior suicidal plans or attempts (CSSRS, Question 3)	−0.01 (−0.08, 0.06)
Three-month prior suicidal plans or attempts (CSSRS, Question 4)	0.10 (0.02, 0.19)
Mental Health	
Distress (K10)	0.02 (−0.05, 0.09)
Depressed mood (QIDS)	0.01 (−0.07, 0.08)
Anxiety (OASIS)	0.03 (−0.04, 0.11)
Psychosis-like experiences (PQ16)	0.04 (−0.04, 0.11)
Manic-like experiences (ASRM)	0.07 (0.00, 0.14)
Suicidal ideation (SIDAS)	0.01 (−0.05, 0.07)
Social support (Schuster’s SSS)	0.06 (−0.01, 0.12)

For the binary variables, we calculate the mean (95% ETI) for the difference in the diffusion parameter for each group.

### Individual trajectory learning and prediction

**Fig. 2:**
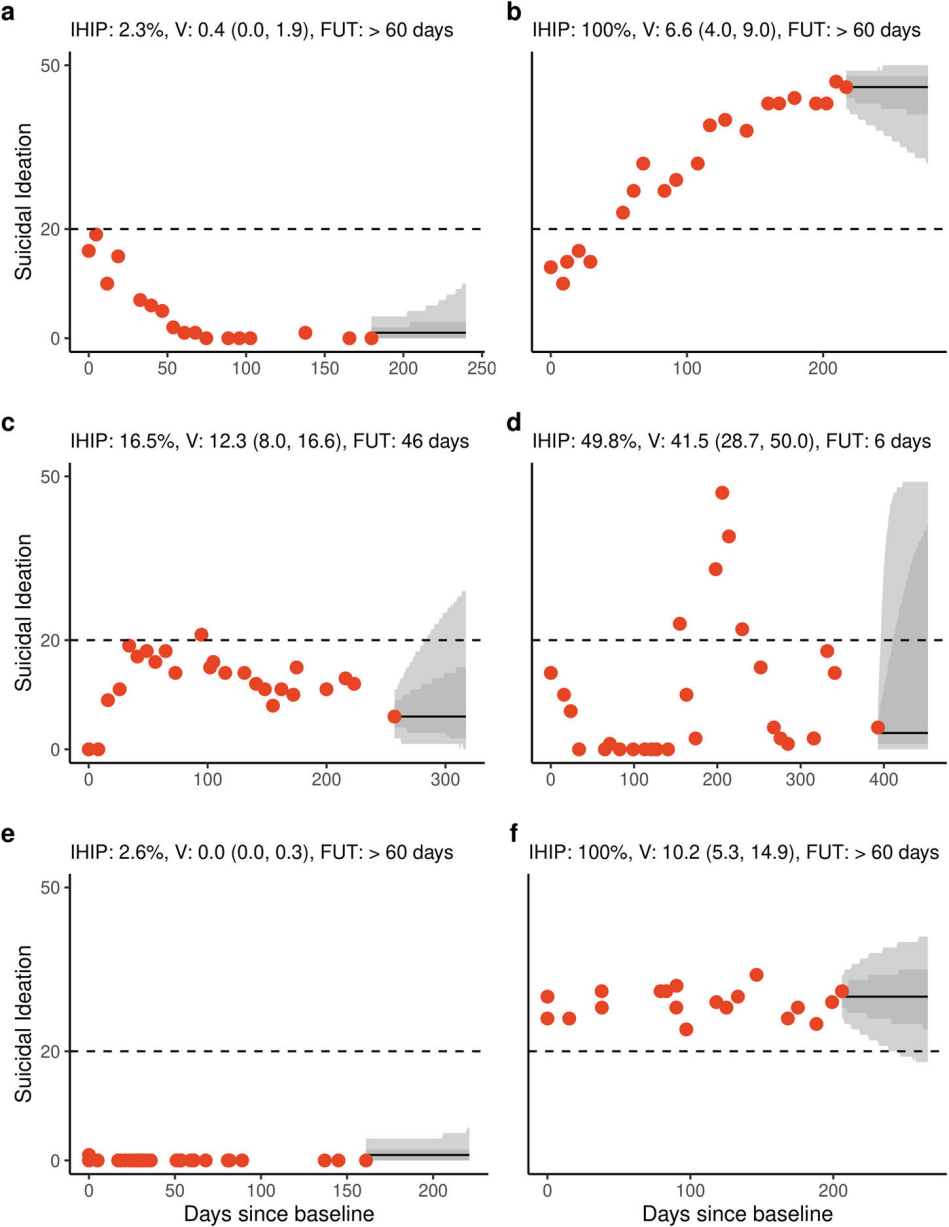
Predictive trajectories for individuals with qualitatively different historical trajectories. The distribution of predicted trajectories 60 days from the last observation for representative individuals that have **a** improved, **b** deteriorated, **c** had moderate risk and volatility, **d** high volatility, **e** no suicidal thoughts, and **f** high risk but moderate volatility. The distribution of trajectories is summarised using the median (black line), 68% (dark grey) and 95% (light grey) ETI each day. To summarise the individual’s future ideation trajectories, we show the probability that the individual will be in the high-ideation category on any day during the next 60 days (integrated high-ideation probability, IHIP), the future observational variability (*V*) and a recommended follow-up time (FUT).

Our focus was on constructing individualised predictions. As such, we show several illustrative examples of individuals within our data. We show six individuals with qualitatively different historical trajectories that are broadly representative of the diversity of trajectories across the population, along with their predicted trajectories from their final observation in Fig. 2. We include historical trajectories that deteriorated (a) and improved (b), as well as those with moderate ideation (c), high variability (d) or moderate variability but with high ideation (f). We also include an individual who had no suicidal ideation while in care (e), which accounts for 152 (26.0%) individuals within our population.

**Fig. 3:**
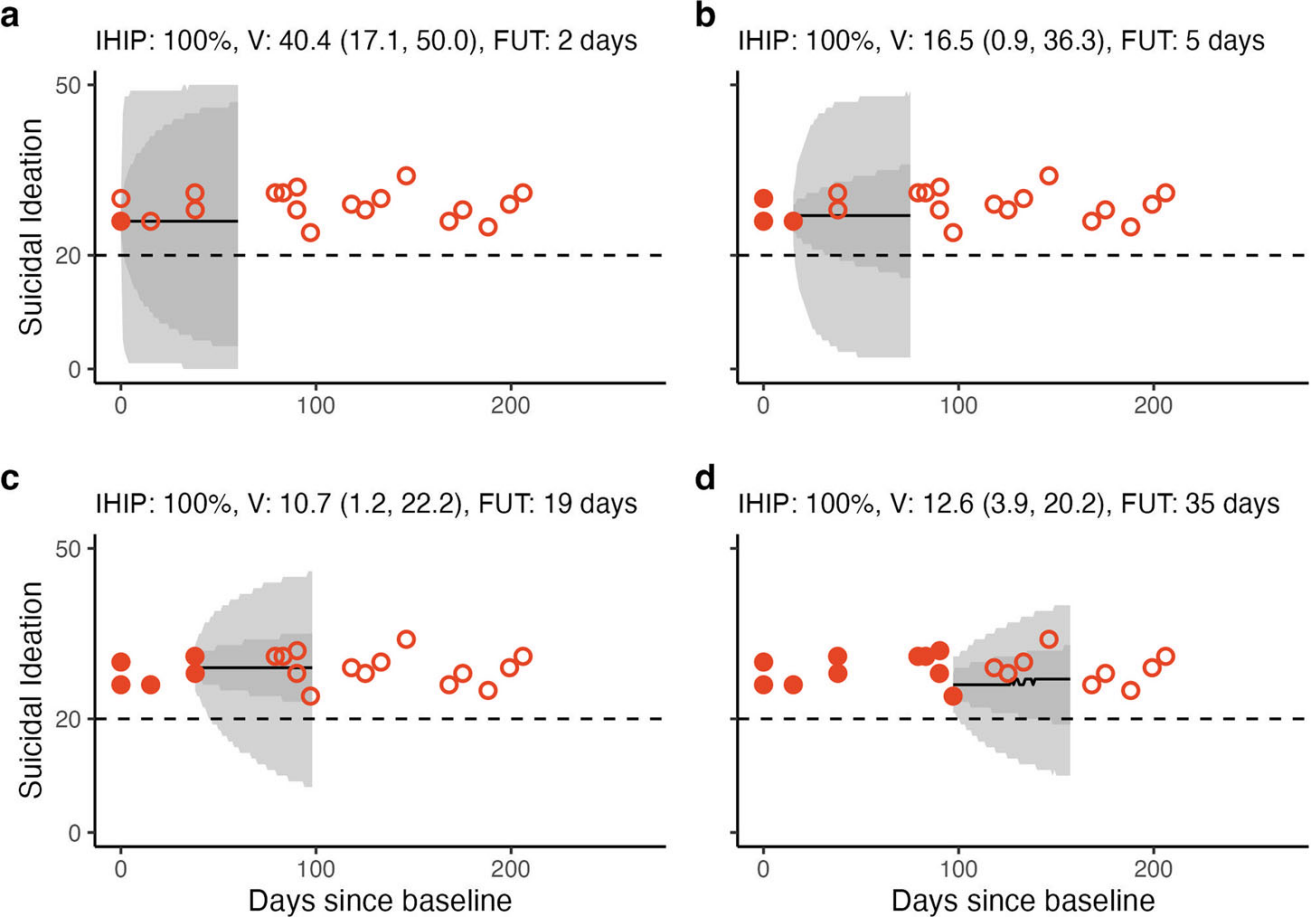
Predictive trajectories for an individual as data is collected. Predictions for 60 days into the future from **a** baseline, and then after **b** three, **c** five, and **d** 10 observations. Each set of predictions uses the observations at or prior to that time (filled red circles) which are compared to future observations (red outlined circles). The distribution of trajectories is summarised using the median (black line), 68% (dark grey) and 95% (light grey) ETI for each day. To summarise an individual’s future ideation trajectories, we show the probability that they will be in the high-ideation category on any day during the next 60 days (integrated high-ideation probability, IHIP), the future observational variability (*V*) and a recommended follow-up time (FUT).

We then show the dynamic process by which predictions are updated for an individual as observations occur. This is an approximation for the process by which individual trajectories would be estimated within a real-world clinical setting as data is collected. This is shown in Fig. 3. We show an individual that has moderate variability in suicidal ideation throughout care. The possible range of trajectories is highly uncertain at baseline (Fig. 3a) but as observations are collected over time (Fig. 3b–d) the ETI ranges decrease, suggesting that the model is learning that this individual has relatively stable suicidal ideation over time.

### Prediction summaries

For each set of predictions, we provide several measures to quantify the level and variability of future suicidal ideation. This includes an estimate of the probability that an individual will be in the high-ideation category over the next 60 days. The variability measure V is an estimate of future observational variability with the distribution of median variability of 10.2 (IQR, 0.0–36.9) across individuals with ≥10 observations calculated from the final observation. We then provide a measure for how long it takes information to be ‘lost’ that we use as a recommended follow-up time (FUT), which has a median of 6 (IQR, 3–19 days) for individuals with ≥10 observations calculated from the final observation.

## Discussion

We constructed continuous-time trajectory models for suicidal ideation using naturalistically collected data in a clinical setting. These dynamic models consider an individual’s current and past observations and population parameters, to learn and update predictions as new observations are collected. We then provided the probability of an individual being in the high-ideation category over a particular time (i.e. IHIP) as well as an estimate for how long it takes to lose information (i.e. FUT). Several insights and applications can be drawn from these analyses.

The best-performing model was the Wiener process model which suggests that the typical trajectory is dominated by variability with no long-term constant. We note that this result will be partly due to assuming that there is one type of model used across the population. Some individuals qualitatively appear to have a stable point, which suggests that a mixture of the two models would be ideal to identify those individuals that have a long-term constant that their trajectories trend towards.

Our work illustrates that there are qualitatively different trajectories of suicidal ideation among those in care. First, our population can be split into a group that had zero suicidal ideation throughout care (26.0%) and ‘suicidal ideators’ who have a wide range of potential trajectories that are poorly known at baseline. This complicates decision-making due to a poor understanding of an individual’s future state, which means that decisions are typically reactive, as opposed to proactive. Despite the high degree of uncertainty about trajectories at baseline, individual trajectories can be learned and predictions can be dynamically updated as more information is collected, which could inform clinicians within MBC settings.

The use of trajectory modelling in MBC settings is still relatively novel, where current approaches tend to predict an expected trajectory by matching an individual’s baseline or latest observations to a subset of the population^25,26^. The comparison between this expected trajectory and an individual’s realised trajectory is used to determine treatment progress and guide clinical decisions. While clearly useful as an approach for MBC, the reliance on an expected trajectory may limit the level of personalisation that could be achieved to effectively tailor interventions to a person’s specific context and history.

An alternative or additional method to using an expected trajectory is to compare an individual’s observations to their prior individual-level predictions. This would allow clinicians to understand when an individual’s observations lie outside their own predicted range of trajectories (i.e. the 68% or 95% ETI). For example, individuals who have suicidal thoughts that emerge with no prior history throughout care will tend to lie outside of the predicted range of trajectories. This could be used as an additional flag for clinicians to understand this emergent phenomenon.

We found that suicidal ideation variability differs across our population. Our model comparison results showed that individualised diffusion parameters were required to model the data. We saw no clear demarcation within this distribution to suggest that there are categorical groups. Instead, we suspect that individuals lie on a continuum of variability, which emphasises that we should estimate trajectories at the individual level.

Differentiating the level of variability is important as it is suspected to lead to different outcomes^48^. It has been argued that those with higher variability have heightened stress responsivity compared to those with lower variability^35,48^. The higher variability individuals may also be more likely to engage in spontaneous suicidal behaviours that would warrant an ongoing need to monitor their care closely and establish personal and social mechanisms to limit such precipitous behaviours. Whereas those that have high and persistent suicidal ideation with low variability may be more likely to engage in suicidal behaviours with greater planning^49,50^. As such, providing clinicians with an understanding of an individual’s variability in suicidal ideation could help to tailor a more personalised suite of interventions.

The frequency of data collection is crucial in the context of MBC and associated tools where data is collected at varying time intervals, ranging from hours (e.g. ecological momentary assessments) or weekly/monthly (e.g. when prompted by a clinician at an appointment). Monitoring and data entry is driven by a multitude of factors including feasibility, ease of collection and motivation and the outcomes of interest (e.g. suicidal ideation, mood) which are important, dynamic and specific to that individual. However, research into the timeliness of data collection for an individual has been limited (see ref. ^51^ as an exception). As such, approaches that can provide timely prompts for information given an individual’s specific circumstances would increase the level of personalised care.

Our analysis highlights that variability differs across the population and that predictions from a baseline observation are highly uncertain. The FUT values indicate how quickly information is lost and provide an approximate measure for how often an individual should be observed. These values tend to be lower when little information about a person is known (e.g. at baseline), or when someone has a highly variable course of illness. This implies that individuals should be observed more frequently during the early stages of care^52^ and when there is high observational variability. For example, the individual in Fig. 2d should be observed frequently (approximately weekly) compared to other individuals that had more stable trajectories.

Decisions regarding monitoring frequency need to consider several modelling and clinical factors not fully accounted for here. The recommendations assume a desired level of certainty (95% ETI range within 50% of the SIDAS range), which can be adjusted to increase or decrease the monitoring frequency. This implies that the FUT estimates should be considered as relative, rather than absolute. Furthermore, the emergence of suicidal ideation within individuals with no prior history of suicidal ideation necessitates ongoing observations. To improve our understanding of these phenomena, future work should focus on allowing for abrupt changes in an individual’s state or incorporating dynamic parameters.

Our suggestion is to observe people when we know that our prior observations have limited predictive value, rather than to observe people at an absolute higher ideation level. This is a product of the random walk nature of the model parameterisation. If there was a dependency between current suicidal ideation scores and the future change in those scores, our recommendations may change.

A future goal for this work is to construct a modelling procedure that could be implemented using digital technologies within an MBC setting. We also want to expand this to several factors that are routinely monitored. The product would be a modelling approach that provides insights about the possible trajectories that an individual could take along with the summarisation of those trajectories—such as the individualised high-ideation probability, variability and recommended FUT—that could provide quick and informative insights for clinicians. We provide an illustrative example of the trajectory updating procedure in Fig. 3.

We acknowledge that there are several limitations in this work. Data are limited to those individuals who have engaged in clinical care using the Innowell platform. Furthermore, the data may not be missing at random as individuals were not obliged to enter their data into Innowell, which could lead to biases in the recorded level of suicidal thoughts.

The model formulation does not fully capture several aspects of suicidal ideation. The most important of which may be the binary aspect of suicidal ideation. Many individuals have no suicidal ideation throughout care, or they may transition from no suicidal ideation to a non-zero value. Similarly, other instantaneous movements or dynamic changes in their diffusion parameters are unlikely to be well-modelled. Model expansions could be made, including transitions to other qualitatively different states^53^ or including time-varying parameters^54^. We also do not explicitly include covariates at baseline within the model.

Model comparison via cross-validation is usually preferred to information criterion measures. However, a $k$-fold cross-validation procedure has a run time on the order of $k$. The sampling procedure typically takes several hours depending on the number of samples requested and the model parameterisation. Thus, testing many different model parameterisations would be infeasible. As such, we used an information criterion approach to allow us to explore models at a faster rate, at the expense of using a more accurate cross-validation technique.

Several simplifications are made to estimate the updated prediction trajectories in Fig. 3. In this case, we fixed the prior for the individual-level parameters using the maximum a posteriori estimates for the population-level parameters. Thus, the population parameter uncertainties are not passed through. This is explained in Supplementary Methods 5.

## Methods

### Ethics

The Northern Sydney Local Health District Human Research Ethics Committees approved this study (HREC/17/HAWKE/480), and all participants gave online informed consent (via an opt-out process).

### Participants

Participants were recruited from a group of individuals who presented for the first time to Headspace services (*N* = 400, 68.4%) in New South Wales, Queensland and South Australia between November 2018 and November 2022. We also include data from several other locations including Mind Plasticity (*N* = 159, 27.1%), Open Arms (*N* = 13, 2.2%), and the Butterfly Foundation (*N* = 13, 2.2%). All individuals presenting to these participating services were deemed eligible for this study, with the added requirement that they could access the Innowell platform. We included all individuals who used the Innowell platform with at least two completed observations for the suicidal ideation measure described below.

### The Innowell platform

The Innowell Platform is an online platform that aims to assist in the assessment, management, and monitoring of mental ill health and maintenance of well-being^3,55^. It is a web-based platform that can be accessed via traditional computing and mobile devices. The platform allows young people to complete web-based clinical assessments to understand their needs; explore their personalised dashboard of results; select from recommended care options (e.g. fact sheets, apps, e-tools and other web-based systems) to support their mental health and well-being; track their progress and share their dashboard and plan with clinicians to support care.

### Measures

After being sent an invitation to join the Innowell platform, participants were asked to complete an initial assessment prior to their scheduled face-to-face appointment with a clinician. All assessment data are part of the functionality of the platform, which participants complete as part of standard clinical care through their service.

The initial clinical assessment covers a range of mental health concerns, as well as comorbid and associated risk factors. The assessment includes a range of biopsychosocial domains, such as mental health (i.e. psychological distress, depressed mood, anxiety, psychosis-like experiences, mania-like experiences and post-traumatic stress), suicidal thoughts and behaviours, social and occupational functioning, sleep-wake cycle, social connectedness, alcohol use, tobacco use, non-suicidal self-harm, physical health, eating behaviours and body image. It also assesses demographic information and a history of physical and mental health problems and treatment.

In addition to the initial questionnaire, individuals can complete a shorter follow-up assessment that included measures of suicidal ideation (SIDAS^56^) and behaviours (C-SSRS^57^), social functioning (SOFAS^58^), overall mental health (Clinical Global Impressions Scale, CGI-S^59^), overall health (single domain on EQ-5D-Y^60^) and social support (Schuster’s Social Support Scale, SSSS^61^). For more details about the measurements available within Innowell see Supplementary Methods 1.

We focus on the summed SIDAS score. SIDAS consists of five questions that focus on suicidal ideation. The questions ask individuals about; their frequency of suicidal thoughts, their ability to control those thoughts, whether they are tormented by those thoughts, and how much their suicidal thoughts impact their daily lives. They are also asked about their recent closeness to an attempt (0 = not close at all, 10 = made an attempt). All questions are asked on a 0–10 scale with higher values being more severe.

The recording of follow-up data was completed along with clinical care. The first summed SIDAS score was taken within the baseline questionnaire. All subsequent assessments are labelled with a time since baseline. As individuals and clinicians were not required to enter data into Innowell the observations can be taken at various times throughout care.

### Statistical analysis

The code for the model parameterisation and sampling scheme was written in Julia (version 1.8). All other analyses were written in R (version 4.3.1).

Our models were built using a data-driven model-building approach, where we started with a simple model parameterisation, and then increased complexity only when model performance warranted it. Such a model-building approach is useful to build simple models that can often have greater predictive performance. However, this model-building approach may leave out important features of the causal processes involved in suicidal ideation, and thus have limited ability in detailing our theoretical understanding of the underlying process.

We used continuous time models to deal with the irregularly time-spaced data. These models assume that the data follows a stochastic differential equation (SDE)^39,40,62^. We explore two simple models known as a Wiener process and an Ornstein–Uhlenbeck process. The Wiener process is the simplest SDE, which corresponds to a continuous time random walk process, meaning that an individual’s ($m$) trajectory will walk away from its current state in a random manner at a rate that is described by a diffusion parameter (${\phi }_{m,2}$). The Ornstein–Uhlenbeck process is the continuous time equivalent of an autoregressive process, and increases the model complexity slightly by adding a deterministic term to the Wiener process that drives an individual’s current state towards a long-term constant value (${\phi }_{m,4}$) at a rate that is described by a drift parameter (${\phi }_{m,3}$). All models have an initial baseline value (${\phi }_{m,1}$) which is a parameterisation of the individual’s initial suicidal ideation.

A novel addition from previous methods used on mental health data is that the modelled state values are converted from an unbounded continuous space to a discretised and bounded score using a logistic link function and a discretised normal distribution. This is implemented using a particle filter method, which allows for the fitting of complex models using simulations when analytical distributions are not available.

We explored several combinations of fixed and random effects for the model parameters. Where random effects are used, those parameters are assumed to follow a normal distribution^42,63^ that describes the population. In this way, the parameterisation allows for both population and individual-level parameters. More details of the model parameterisation can be found in Supplementary Methods 2.

The model was estimated within a Bayesian framework via posterior sampling^64^. The aim of these techniques is to sample from the posterior distribution $p\left(\theta ,|,y\right)\propto p(\theta )p({y|}\theta )$, where $\theta$ is a set of parameters of interest that we would like to learn given some data $y$. The right-hand side informs us that our posterior knowledge is a function of our prior knowledge encoded in $p(\theta )$ which is updated with respect to the likelihood $p({y|}\theta )$. We construct a scheme to sample from the posterior distribution that builds on the work by Wiqvist et al.^65^. This sampling scheme uses a correlated pseudo-marginal metropolis-hastings scheme to sample the individual parameters within a set of population parameters. The population parameters are then sampled using a semi-conjugate Gibbs sampling scheme. More details of this procedure are provided in Supplementary Methods 3.

Trajectories are a summary of the posterior distribution, where we use the median and equal-tailed credible intervals (ETI^66^, a Bayesian equivalent to confidence intervals) as descriptors.

Predictions are constructed using the posterior predictive distribution. This involves simulating future trajectories many times while iterating over the parameter values drawn from the posterior distribution. More details on this procedure are provided in Supplementary Methods 4.

In all cases, we estimate the posterior predictive distributions for 60 days into the future from a reference observation. Sixty days was chosen as a reasonable period which was consistent with our typical time difference between observations, but it could be changed to account for other time periods of interest. We then summarise the distribution by calculating the median and the ETI on each day. We also provide two severity-related statistics to summarise the posterior predictive trajectories. We calculate the probability that the individual will be in the high-ideation category (SIDAS > 20) on any day over the next 60 days (i.e. IHIP). Furthermore, we calculate the number of days that it would take for the 95% ETI to encompass at least 50% of the SIDAS range (i.e. 95% ETI range > 25) as an estimate for how long it takes to ‘lose’ information given the previous observations. We refer to this as the FUT throughout the paper. We also provide a measure of future observational variability (*V*), which is the expected ETI range 60 days from a baseline observation, along with the 80% highest density credible intervals (HDI) for that ETI range. We considered the 80% HDI range for V to be more parsimonious than wider measures as the distribution can be very wide-tailed and asymmetric. These summaries aim to provide a snapshot of the possible trajectories that an individual could take as well as their overall severity.

### Model comparison

As a statistical model comparison tool, we compare the predictive ability of different models using the WAIC^67^. The WAIC is similar to the Akaike information criteria as it estimates the out-of-sample predictive accuracy using a combination of the in-sample log-likelihood and a punishment for the degrees of freedom of the model^46,47^. However, the WAIC is appropriate for Bayesian models where we have the posterior distribution of the log-likelihood rather than a maximum log-likelihood estimate. Furthermore, in hierarchical models, the degrees of freedom are typically less than the total number of parameters. The degrees of freedom for the WAIC are estimated as the variance of the posterior log-likelihood. WAIC is calculated on the deviance scale, and thus models with lower WAIC are preferred. While cross-validation methods provide a more robust measure of out-of-sample predictive accuracy, we considered such methods to be too computationally costly in our case.

### Supplementary information

Supplementary information

## Data Availability

The datasets used and analysed during the current study are available from the corresponding author upon reasonable request.

## Code availability

All relevant code for this paper can be found at github.sydney.edu.au/mvar0005/dynamiclearning/.
