# Supplementary material for: Dynamic learning of individual-level suicidal ideation trajectories to enhance mental health care
Source: Npj Ment Health Res. 2024 Jun 7;3:26. doi: 10.1038/s44184-024-00071-0 (PMC11161660; doi:10.1038/s44184-024-00071-0)
Supplement: Supplementary file 1 — Supplementary information [file 44184_2024_71_MOESM1_ESM.pdf]

Supplementary Methods 1.

The following provides detailed information with respect to the questionnaires provided on the Innowell platform and was adapted from Capon, et al.[1].

Overall health

EQ-5D-Y – The EQ-5D-Y (Youth) is a questionnaire that measures the generic health-related quality of life[2]. The Innowell platform only uses a single question related to an individual's health, which asks "How good is your health TODAY? Please respond using a number between 0 (the worst health you can imagine) and 100 (The best health you can imagine)." The individual is given a 100-point scale from which they can select their answer.

Overall mental health

CGI-S – The Clinical Global Impressions – Severity scale measures an individual's current mental health. It asks a single question; "How would you rate your mental health at this time?". The individual has a selection of 7 items that range from "Normal, not at all unwell" (1) to "Extremely unwell" (7).

Suicidality

SIDAS – The Suicidal Ideation Attributes Scale (SIDAS) is a five-item scale assessing suicidal ideation over the past month. The scale assesses frequency, controllability, closeness to attempt, distress and interference with daily activities on a 10-point Likert scale (ranging from 0 ("never") to 10 ("always")). A total score of zero corresponds to "no current ideation", a score of one to 20 corresponds to "low current suicidal ideation", and a score of 21 to 50 corresponds to "high current suicidal ideation". The scale has strong internal reliability (Cronbach $\alpha = 0.91$)[3].

C-SSRS – The Columbia-Suicide Severity Rating Scale (C-SSRS) aims to measure suicidal thoughts and behaviours in the past month and entire lifetime[4]. The questionnaire assesses four constructs: severity of ideation, intensity of ideation, suicidal behaviours, and lethality.

Distress

K-10 – The Kessler-10 (K-10) measures psychological distress over the past 4 weeks[5]. It is a well-validated measure widely used in adult and adolescent populations in both clinical and community settings. The scale consists of 10 items with five multiple choice options ranging from "none of the time" (1) to "all of the time" (5). Total scores range from 10-50. A total score of under 20 indicates 'likely no distress', 20-24 indicates "likely mild mental disorder", 25-29 indicates "likely moderate mental disorder", over 30 indicates 'likely severe mental disorder'[6]. The K-10 has moderate reliability (kappa scores 0.42-0.74).

Psychosis-like experiences

PQ-16 – The Prodromal Questionnaire (PQ-16) is a self-report measure used to screen for individuals at risk of psychosis and select individuals for interview of psychosis risk[7]. It was adapted from the 92-item Prodromal Questionnaire, including nine items from the perceptual abnormalities subscale, five items from the unusual thought content/delusional ideas subscale, and two items from the negative symptoms subscale[8]. Questions require "True" or "False" answers to statements describing feelings, experiences, or symptoms of psychosis. In

the event of a "True" response, the participant is required to then interpret the perceived distress of that on a Likert scale (0-3, with 3 being severe distress). A cut-off score of 6 or more on the symptom-scale has been shown to detect at-risk mental states with 87% specificity and sensitivity[7].

Mania-like experiences

ASRM – The Altman Self-Rating Mania Scale (ASRM) is a 5-item scale used to assess the presence and/or severity of manic-like symptoms during the past 7 days. Each item is scored on a 5-point scale (0-4), with total scores ranging from 0-20 and higher scores indicating greater severity. A score greater than 5 indicates a high likelihood of a manic or hypomanic condition, as per 86% sensitivity and 87% sensitivity[9].

Functioning

Youth not in education or employment (NEET) – Engagement with either employment, education, or training was based on questions from the Organisation of Economic Co-operation and Development (OECD), and Census of Population and Housing, Australian Bureau of Statistics (ABS)[10]. These four multiple choice questions were: 1) Are you currently engaged in education or study (e.g. school, TAFE or university) on a regular basis?; 2) Are you currently engaged in paid employment or work on a regular basis?; 3) Are you currently engaged in voluntary work through an organisation or group on a regular basis?; and 4) Are you currently providing unpaid care, help or assistance to family members or others because of a disability, a long term illness or problems related to old age on a regular basis? Individuals not involved in employment, education, or training were classified as NEET.

WSAS – The Work and Social Adjustment Scale (WSAS) is a brief and reliable measure of work and social adjustment. The questions aim to assess whether an individual is currently impaired or unable to perform day-to-day tasks, due to their mental health. The scale consists of five items that require the individual to rate between 0-8 ("not at all" to "very severely"), based on their agreement with the statement. A maximum total score is achieved by summing all 5 items. A total score greater than 20 suggests moderately severe psychopathology, 10-20 suggests significant functional impairment with less severe symptomatology, and scores under 10 are associated with subclinical populations. The scale has a test-retest correlation of 0.73 and has correlations of 0.76 for severity of depression and 0.61 for obsessive-compulsive disorder symptoms[11].

SOFAS – The Social and Occupational Assessment Scale is a single question that asks an individual to rate their social and occupational functioning[12]. The question on the Innowell platform asks, "Thinking about your ability to participate in everyday social, school (including university/college) or work activities, how much of your "usual activities have been limited?". The individual is then allowed to select 7 options that range from "Only everyday problems or concerns (e.g., mild anxiety before an exam or an occasional argument with family members)" (1) to "Inability to participate in almost all areas of life (e.g., stay in bed all day, or no job, home or friends or think about harming yourself)" (7).

Alcohol, tobacco, cannabis use

ASSIST – The Alcohol, Smoking and Substance Involvement Screening Test (ASSIST) is an 8-item questionnaire that aims to detect substance use-related problems. It screens for use of

tobacco, alcohol, cannabis, cocaine, amphetamine-type stimulants, sedatives and sleeping pills, hallucinogens, inhalants, opioids, and other drugs. Besides the first question which concerns life-time experiences, each question requires the individual to respond to questions that concern the prior 3-months on either 5- or 3- point Likert scales[13].

AUDIT-C – The Alcohol Use Disorders Identification Test (AUDIT-C) is a brief measure consisting of three questions related to the frequency of general and binge alcohol consumption over the past year[14]. Each question has 5 options that increase in frequency or quantity of consumption (scores range from 0-4)[15]. A total score of 0-3 indicates low-risk drinking, 4-5 moderate risk, and scores greater than five indicates high risk drinking, however, this may not apply if total points come from q1 (i.e., when q2 and q3 =0).

Social connection
SSSS – The Schuster's Social Support Scale (SSSS) is a 6-item questionnaire that aims to assess the frequency of both supportive and negative interactions with family and friends[16]. The first five questions are scored on a 4-point Likert scale (0-3; "never" to "often") with alpha reliability ranging from 0.56-0.75, and the sixth question requires a Yes/No response[16].

Depression
QIDS-SR – The Quick Inventory of Depressive Symptomatology – Self-report (QIDS-SR) consists of 16-items that aim to assess nine domains of depression during the preceding seven days[17]. These domains are related to the DSM-IV diagnosis of a major depressive disorder, including: sleep, mood, appetite/weight, concentration/decision making, self-view, suicidal ideation, general interest, energy level, and agitation. Each item is scored on a scale between 0-3 points and scoring instructions determine the total score (which ranges from 0-27)[18]. Scores greater than 21 indicate very severe depression, 16-20 indicate severe depression, 11-15 indicate moderate depression, 6-10 indicate mild depression, and scores of 5 or lower indicate no depression.

Anxiety
OASIS – The Overall Anxiety Severity and Impairment Scale aims to assess the severity and impairment of anxiety-related symptoms over the past seven days[19]. The scale consists of five multiple choice questions with five options that are scored from 0-4, with higher scores indicating greater severity and/or impairment. A cut-off score of 8 has high validity (87%) for identifying anxiety disorders[20].

Physical health
Height, weight, and waist circumference
Body mass index (BMI) is calculated by Weight/(Height^2) to estimate total body fat in proportion to total body weight. It is used to estimate risk of cardiovascular, metabolic, and other diseases. Waist circumference, however, is a more accurate estimate of visceral fat and more predictive of cardiovascular diseases. For women, a waist circumference of 80-87cm is considered increased risk, and 88+cm is greatly increased. For men, a waist circumference of 94-101cm is considered increased risk, and 102+cm is considered greatly increased risk[21].

IPAQ – The International Physical Activity Questionnaire (IPAQ) aims to determine the average physical activity of an individual and can be scored on a continuous and/or categorial

scale (high, moderate, or low)[22]. First, all activity is calculated in minutes. Second, minutes should be converted to metabolic equivalent of task (MET) minutes (multiply the minutes by the relevant scalar; walking =3.3, moderate activity=4, vigorous activity =8). Third, multiply MET minutes by number of days the activity was performed. For categorical scoring, high = over 3000 MET minutes a week OR over 1500 MET minutes per week with 3 or more days of vigorous exercise; moderate = at least 3 days of vigorous activity or walking of 30 or more mins per day, OR 5+ days of moderate intensity activity and/or walking (minimum 30 mins per day), OR 5+ days of walking, moderate intensity, or vigorous activity equating to 600+ MET minutes; and low = not meeting high or moderate.

Sleep-wake cycle
PSQI – The Pittsburgh Sleep Quality Index (PSQI) is a questionnaire that aims to assess sleep quality and disturbances over the past month[23]. It consists of nineteen-items that assess seven domains: sleep quality, sleep latency, sleep duration, habitual sleep efficiency, sleep disturbances, use of sleeping medication, and daytime dysfunction. Each of the seven component scores are summed together to form a global score[23].

MCTQ – The Munich Chronotype Questionnaire (MCTQ) is a self-report scale that assesses bed- and rise-times, and self-assessment of individual chronotype[24]. The chronotype options range from extremely early to extremely late and is determined by using the midpoint between onset and offset of sleep.

Post-traumatic stress
PC-PTSD-5 – The Primary Care PTSD Screen (PC-PTSD) is a five-item questionnaire that aims to assess PTSD symptoms which reflect the DSM-V diagnostic criteria[25]. Each item is scored as either Yes or No (1 or 0), and a maximum total score is 5. A cut-off of 3 is optimally sensitive (reduces false negatives; $\kappa[1]$) = 0.93, standard error = 0.041), yet a cut-off of 4 is optimally efficient (good balance between false positive and negatives; $\kappa[0.5] = 0.63$, standard error = 0.052)[25].

Eating behaviours and body image
EDE – The Eating Disorder Examination Questionnaire (EDE-Q) is based on the eating disorder examination interview and aims to assess eating behaviours and body image disturbance over the past four weeks[26]. The questionnaire uses a combination of Likert scales and Yes/No options.

Supplementary Methods 2.

The following provides supplementary information with respect to the model parameterisation.

Notation

We use the following notation throughout this supplement:

- Random variables are denoted by capital letters with their realisations denoted by lowercase.
- Bold font to represent vectors.
- Underline plus bold face to indicate a set of vectors.
- As short hand we define $\boldsymbol{s}_{c:d} = \{s_a, s_{c+1}, s_{c+2}, \dots, s_d\}$ for $d > c$. We also use a similar notation for t where $\boldsymbol{s}_{t_a:t_b}$ means all realisation in the time interval from t_a to t_b inclusive of t_a and t_b.
- Superscript asterisks are used for proposed parameters.
- Tilde accents are used for predictive variables.

State space models

Consider that we want to model the suicidal ideation trajectories for an individual m. Our data is a sum of five questions each on a 10-point scale, leading to a total score that is discretised and bounded to the range $[a = 0, b = 50]$. The observations can also be observed at any future time given by t from a baseline observation at time $t = 0$. This motivated us to use state space models. State space models consist of two processes; one of which describes the underlying latent state $\{X_{m,t} \in \mathbb{R} | t \geq 0\}$ and another that links the latent state to an observed state $\{Y_{m,t} \in \mathbb{Z} | a \leq Y_{m,t} \leq b, t \geq 0\}$.

Continuous time models

We assume that the latent states follow a continuous time model which is described by a stochastic differential equation (SDE[27]). We consider two possible SDEs known as the Wiener and Ornstein-Uhlenbeck processes. Both of these models can be used to simulate realisations for a future latent state $x_{m,t+\Delta t}$ given the current realised state $x_{m,t}$, the time interval Δt, a set of parameters $\boldsymbol{\theta}_m$, and a particle that follows $u_{m,t,i} \sim N(u_{m,t,i};\ 0, 1)$ using a transition function $f_{.}(x_{m,t+\Delta t} | x_{m,t}, \boldsymbol{\theta}_m, u_{m,t,i})$.

The Wiener process is the continuous time equivalent to a random walk process. The discretised realisation of this continuous time process is,

$$f_{\mathrm{W}}\left(x_{m,t+\Delta t} | x_{m,t}, \boldsymbol{\theta}_m, \boldsymbol{u}_{m,t}\right) = x_{m,t} + u_{m,t,i}\theta_{m,2}\sqrt{\Delta t} \quad (1)$$

,

$$x_{m,0} | \boldsymbol{\theta}_m = \theta_{m,1}$$

The second term is a stochastic component that describes how far away the future state value can lie from its current value (also referred to as diffusion throughout the paper).

The Ornstein-Uhlenbeck process is the continuous time equivalent to an autoregressive process. The discretised realisation of this continuous time process is,

$$f_{\text{ou}}\left(x_{m,t+\Delta t}\middle|x_{m,t},\boldsymbol{\theta}_m,\boldsymbol{u}_{m,t}\right) = \theta_{m,4} + \left(x_{m,t} - \theta_{m,4}\right)e^{-\theta_{m,3}\,\Delta t} + u_{m,t,i}\theta_{m,2}\sqrt{\frac{1}{2\theta_{m,3}}\left(1 - e^{-2\theta_{m,3}\Delta t}\right)} \quad (2)$$

$$x_{m,0}|\boldsymbol{\theta}_m = \theta_{m,1}$$

This extends the Wiener process by adding a deterministic component that is a function of the difference between an individual's current state and a long-term constant $\theta_{m,4}$. When $\theta_{m,3} > 0$ this process drives the latent state towards the long-term constant ensuring a stationary process. If $\theta_{m,3} < 0$ then $x_{m,t} = \theta_{m,4}$ is an unstable equilibrium point and any deviation from that value will lead the latent state to be driven to ±infinity.

Linking procedure

The latent state is then linked to an observed state that is qualitatively consistent with our data by a procedure given by $g(y_{m,t}|x_{m,t},\sigma_\epsilon^2)$ where we assume that the observations follow a discrete and bounded normal distribution with a variance of σ_ε^2. The probability that an individual has an observation $Y_{m,t} = y_{m,t}$ is then given by,

$$g\left(y_{m,t}\middle|x_{m,t},\sigma_\epsilon^2\right) = \frac{\int_{\max\left(a,k-\frac{1}{2}\right)}^{\min\left(b,k+\frac{1}{2}\right)} N\left(y_{m,t};h\left(x_{m,t}\right),\sigma_\varepsilon^2\right)}{\int_a^b N\left(y_{m,t};h\left(x_{m,t}\right),\sigma_\varepsilon^2\right)} \quad (3)$$

where $N(k;h(X_{m,t}),\sigma^2 = \sigma_\varepsilon^2)$ is the normal probability density function calculated at k where the mean value is given by the logit link function,

$$\mu_{m,t} = h\left(X_{m,t}\right) = a + \frac{b-a}{1 + e^{-X_{m,t}}}. \quad (4)$$

The choice of linking procedure has been pre-supposed. Many linking procedures are possible, and may even be improvements on the current procedure, but have been left out of the scope of this paper.

We also assumed the observational variance value σ_ε^2. It is possible to make this a random variable which is also fitted. However, we found it difficult for this to converge to sensible values across multiple chains, where it would either converge to an infinitesimally small or extremely large value. We did explore changing the priors in the hope of fitting this more consistently but found that it was not possible unless we set a highly restrictive prior. As such, we set this to half an integer squared per item, then summed across multiple item questionnaires. The summed score has five questions, and therefore, we assume the variance

is $\sigma_\varepsilon^2 = 2.5$. We leave σ_ε^2 out of most of the discussion and notation below for simplification reasons.

The full state space model

Summarising the above, the probability distribution for a future observation given its current state is given by,

$$p(y_{m,t+\Delta t}, x_{m,t+\Delta t} | \boldsymbol{\theta}_m) = g(y_{m,t+\Delta t} | x_{m,t+\Delta t}) f_{.}(x_{m,t+\Delta t} | x_{m,t}, \boldsymbol{\theta}_m, u_{m,t,i}), \quad (5)$$

and the initial observation is given by

$$p(y_{m,0}, x_{m,0} | \boldsymbol{\theta}_m) = g(y_{m,0} | x_{m,0}) \delta(x_{m,0} - \theta_{m,1}), \quad (6)$$

where $\delta(x_{m,0} - \theta_{m,1})$ is the dirac-delta function peaking at $x_{m,0} = \theta_{m,1}$.

Unbounded parameters for fitting purposes

The parameters for an individual are shown above as $\boldsymbol{\theta}_m$. We prefer to parameterise in the unbounded space and then convert to the bounded space, as such we introduce the parameters $\boldsymbol{\phi}_m$. For the Wiener process we then have $\boldsymbol{\theta}_m = \{\phi_{m,1}, \exp(\phi_{m,2})\}$ which ensures a positive diffusion rate as $\theta_{m,2} > 0$. For the Ornstein-Uhlenbeck process we use $\boldsymbol{\theta}_m = \{\phi_{m,1}, \exp(\phi_{m,2}), \exp(\phi_{m,3}), \phi_{m,4}\}$ which ensures stationarity by enforcing $\theta_{m,3} > 0$. We label the function that performs the mapping $\boldsymbol{\phi}_m \to \boldsymbol{\theta}_m$ as $q_{.}(\boldsymbol{\theta}_m | \boldsymbol{\phi}_m)$.

Hierarchical modelling

Now, we want to model the full set of M individuals and share information across the individuals in an hierarchical manner. This requires a probability distribution for the individual parameters that is constrained by the population. As such, we introduce a set of population parameters $\boldsymbol{\eta} = \{\mu_j, \tau_j \mid \forall\, j \in \boldsymbol{J}_\eta\}$ where $\boldsymbol{J}_\eta$ are the indices for the set of parameters that are dealt with as random effects. Then an individual-level parameter $\phi_{m,j}$ will follow a normal distribution with population level mean μ_j and precision τ_j, such that

$$\phi_{m,j} \sim N(\phi_{m,j};\ \mu_j,\ \sigma^2 = \tau_j^{-1}). \quad (7)$$

The population level parameters are estimated simultaneously with the following priors,

$$\mu_j \sim N\left(\mu_j;\ \mu_{0_j},\ \sigma^2 = (M_{0_j} \tau_j)^{-1}\right), \quad (8)$$

$$\tau_j \sim Ga(\tau_j;\ \alpha_j,\ \text{rate} = \beta_j). \quad (9)$$

For our problem we assume the hyperparameters,

$$(\mu_{0,1}, M_{0,1}, \alpha_1, \beta_1) = (0,1,1.5,0.2),$$
$$(\mu_{0,2}, M_{0,2}, \alpha_2, \beta_2) = (-2,1,2,1),$$
$$(\mu_{0,3}, M_{0,3}, \alpha_3, \beta_3) = (-2,1,2,1),$$
$$(\mu_{0,4}, M_{0,4}, \alpha_4, \beta_4) = (-2,1,2,1).$$

In a Bayesian context placing priors on the population level distributions as above is required. The normal-gamma prior for the population level parameters was chosen as it is

the semi-conjugate prior to the normal individual-level parameter distribution when we have unknown mean and variance. For the baseline parameter, we assume $\alpha_1 = 1.5$ and $\beta_1 = 0.2$, which allows the population level precision to be in the range (0, 28] with 99% probability, and for the remaining parameters we assume $\alpha_j = 2$ and $\beta_j = 1$, which allows for the population-level precision to be within the range (0, 7] with 99% probability, both of which are quite diffuse priors allowing for a wide range of individual-level variation. We allowed the precision uncertainty to be larger for the baseline parameter as those values are well constrained by the initial observation, whereas the remaining parameters are less well known, and thus we enforce slightly more prior information. We then assume that the population mean for the baseline parameters follows a standardised normal distribution, which assumes that the expected baseline observation falls in the range [2, 48] with 99.7% probability. The remaining parameters have their prior for the population mean shifted down by -2. Subtracting by two allows for the log of the population level mean drift and diffusion to be in the range (-5, 1) corresponding to [0.007, 2.718] after exponentiation with 99.7% probability. These ranges were chosen after simulating trajectories with various values for the drift and diffusion. Allowing the drift and diffusion rates to be greater than this leads to nearly instantaneous change, whereas below this allows rates of change that are approximately zero leading to no convergence towards the long-term constant. The population mean for the constant is then allowed to vary between [0.3, 36] in observational space with 99.7% probability, which allows for a potential treatment effect compared to the distribution for the baseline values. We note that we did explore varying these hyperparameters, but didn't find any significant change in the posterior distribution of results when doing so.

Fixed effects

We introduce an extra vector of parameters $\boldsymbol{\kappa} = \{\kappa_j | \forall\, j \in \boldsymbol{J}_\kappa\}$ where $\boldsymbol{J}_\kappa$ are the indices for the set of parameters that are dealt with as fixed effects. We set $\phi_{m,j} = \kappa_j$ for all m when the j-the parameter is treated as a fixed effect. The prior for the fixed population parameters is set to $\kappa_j \sim N(\kappa_j\, ;\, 0,\, \sigma_j^2 = 5^2)$ for all j treated as fixed effects.

Supplementary Methods 3.

Particle filters

Particle filters are useful for the continuous time state space models described above as they allow for the estimation of the log-likelihood for (particularly non-linear) stochastic processes[28]. They do this by randomly sampling a vector of particles for each time given by,

$$\boldsymbol{u}_{m,t} = \{u_{m,t,i}\}_{i=1}^{d_m} \tag{10}$$

where d_m is the number of particles for individual m, and this follows a multivariate normal distribution given by,

$$z(\boldsymbol{u}_{m,t}) = N(\boldsymbol{u}_{m,t};\ 0,\ I_{d_m}) \tag{11}$$

with I_{d_m} being the identity matrix of corresponding size. Then the simulation step is performed across all observations, using those simulations to estimate the log-likelihood per particle, and marginalising out the particles by performing a weighted average over those log-likelihood estimates. Given enough particles this is an unbiased and low variance estimator of the log-likelihood. We note that in detail, $d_m - 1$ particles are used explicitly to simulate trajectories, with one particle used for systematic resampling of the $d_m - 1$ particles used for trajectory simulation[29], which provides a better estimate for the log-likelihood, but we won't delve further into that detail throughout this work.

Pseudo-marginal Metropolis-Hastings (PMMH) algorithms use particle filters for their log-likelihood estimator within a Markov chain Monte Carlo (MCMC) scheme[30]. As these algorithms require a reliable estimate of the log-likelihood function, if there are too few particles resulting in high variance in the log-likelihood estimate, these algorithms can often get stuck for many iterations after an over-estimate of the log-likelihood in a prior iteration. We found that this problem was an issue for our data. We initially used the R package nimble/nimbleSMC[31], which implements PMMH algorithms for hierarchical models but found that it would typically get stuck even with a large number of particles. As such, we looked to other methods.

Particle filters are particularly useful for non-linear and non-normal stochastic processes. As we are making several normality assumptions we would likely get similar results using a variation on Kalman filters[32]. In our case, using an extended Kalman filter where we assume that the latent state is linked to the observation space using a logistic function and then truncating the distribution for the observations to the bounded space as a postprocessing step would be possible. Instead, we've decided to deal with the boundaries and discretisation within the fitting process using particle filters. We also hope that this is an initial step in building models that have more complex processes (e.g., jumps, stochastic volatility) that would require particle filters.

Correlated particle filters

Correlated particle filter methods have been developed to deal with some of the problems that occur in particle filters[33–35]. The key to correlated particle filters is first to identify that the discretised realisation of the SDEs described previously are deterministic given a vector of particles $\boldsymbol{u}_{m,t}$. Sampling schemes using correlated particle filters leverage this property by incorporating them as random variables that become part of the sampling procedure. Then the particles follow a proposal distribution with initial values drawn from,

$$z(\boldsymbol{u}_{m,t}) = N(\boldsymbol{u}_{m,t};\ 0,\ I_{d_m}) \quad (12)$$

and subsequent draws from the proposal distribution given by,

$$K(\boldsymbol{u}^*_{m,t}|\boldsymbol{u}_{m,t}) = N(\boldsymbol{u}^*_{m,t};\ \rho\boldsymbol{u}_{m,t}, (1-\rho^2)I_{d_m}), \quad (13)$$

where I_{d_m} is the identity matrix of size equal to the number of particles d_m in the vectors $\boldsymbol{u}_{m,t}$ and $\boldsymbol{u}^*_{m,t}$. The value ρ is the degree of correlation between $\boldsymbol{u}_{m,t}$ and $\boldsymbol{u}^*_{m,t}$ which we set to 0.999. Correlating the particles across iterations acts to reduce the variance in the difference in the log-likelihood which appears in the Metropolis-Hastings algorithm, thus allowing the sampling scheme to be more efficient.

Given the full set of random effects $\underline{\boldsymbol{\phi}} = \{\boldsymbol{\phi}_m\}_{m=1}^{M}$, particles $\underline{\boldsymbol{u}} = \{\boldsymbol{u}_{m,0:T_m}\}_{m=1}^{M}$ where T_m is the time for each individual's final observation, and data $\underline{\boldsymbol{y}} = \{\boldsymbol{y}_{m,0:T_m}\}_{m=1}^{M}$, the posterior distribution that we would like to sample from is given by, $p\left(\underline{\boldsymbol{\phi}}, \boldsymbol{\kappa}, \boldsymbol{\eta}, \underline{\boldsymbol{u}} \middle| \underline{\boldsymbol{y}}\right)$. Following Wiqvist et al.[34] we split this up into the following blocks;

1. $p(\boldsymbol{\phi}_m, \boldsymbol{u}_{m,0:T_m}|\boldsymbol{\eta}, \boldsymbol{y}_{m,0:T_m}) \propto p(\boldsymbol{\phi}_m|\boldsymbol{\eta})p(\boldsymbol{y}_{m,0:T_m}|\boldsymbol{\phi}_m, \boldsymbol{\kappa}, \boldsymbol{u}_{m,0:T_m})z(\boldsymbol{u}_{m,0:T_m})$ for $m = 1, \dots, M$
2. $p\left(\boldsymbol{\kappa} \middle| \boldsymbol{\eta}, \underline{\boldsymbol{\phi}}, \underline{\boldsymbol{y}}, \underline{\boldsymbol{u}}\right) \propto p(\boldsymbol{\kappa}) \prod_{m=1}^{M} p(\underline{\boldsymbol{y}}|\boldsymbol{\phi}_m, \boldsymbol{\kappa}, \boldsymbol{u}_m)$
3. $p\left(\boldsymbol{\eta} \middle| \underline{\boldsymbol{\phi}}, \underline{\boldsymbol{y}}, \underline{\boldsymbol{u}}\right) = p(\boldsymbol{\eta}|\boldsymbol{\phi}_{1:M}) \propto p(\boldsymbol{\eta})\prod_{m=1}^{M} p(\boldsymbol{\phi}_m|\boldsymbol{\eta})$

Steps 1 and 2 are implemented using a Correlated Pseudo-Marginal Metropolis-Hastings (CPMMH) step. Step 3 is implemented using a Gibbs sampler. Further motivation for the block formulations are discussed by Wiqvist et al.[34] and similarly by Botha, Kohn, & Drovandi[35] who explore other procedures.

Code for the sampling scheme is implemented in the Julia language (v1.8). The code began as a fork from the repository used by Wiqvist et al.[34]. The main changes to the code include; 1) allowing for a different number of particles per individual, 2) compartmentalising the model parameterisation from the sampling scheme, such that we can use the same sampling code across multiple models, and 3) minor refactorization to improve readability and understanding of the code to the authors.

Number of particles

Choosing the number of particles for any particle filter method is a fundamental problem[36]. Too many particles can significantly slow the code. Whereas too few particles leads to

increased variance and bias of the log-likelihood estimator, which can lead to inefficient or inaccurate sampling of the posterior distribution, as discussed previously.

The number of particles required typically increases with the number of observations and complexity in the trajectories. In our case, the number of observations differs per individual. Trajectories are also qualitatively different with different degrees of fluctuations that require a differing number of particles. As such, the optimum number of particles is different per individual. We broadly follow the recommendations by several authors[34,36] to estimate the number of particles required per individual.

We start by running four 'short' chains of 10000 iterations with $D = 1000$ particles for all individuals and use the maximum a posteriori (MAP) as a single point estimate. The parameters of this point estimate are then used to estimate the variance and correlation of the total log-likelihood $p(\underline{\boldsymbol{y}}|\underline{\boldsymbol{\phi}},\boldsymbol{\kappa},\underline{\boldsymbol{u}}) = \prod_{m=1}^{M} p(\boldsymbol{y}_m\,|\boldsymbol{\phi}_m,\boldsymbol{\kappa},\boldsymbol{u}_m,\sigma_\epsilon^2)$. This is done by; 1) sampling the particles in accordance with their initial proposal $z(\boldsymbol{u}_{m,t})$ for all t and m and estimating the log-likelihood $p(\underline{\boldsymbol{y}}|\underline{\boldsymbol{\phi}},\boldsymbol{\kappa},\underline{\boldsymbol{u}})$, 2) sampling a subsequent set of particles in accordance with the proposal $K\left(\boldsymbol{u}_{m,t}^{*}\middle|\boldsymbol{u}_{m,t}\right)$ for all t and m then estimating the log-likelihood $p(\underline{\boldsymbol{y}}|\underline{\boldsymbol{\phi}},\boldsymbol{\kappa},\underline{\boldsymbol{u}}^{*})$, 3) repeating steps 1-3 100 times to create vectors of estimated log-likelihood values, and 4) calculating the correlation between $p(\underline{\boldsymbol{y}}|\underline{\boldsymbol{\phi}},\boldsymbol{\kappa},\underline{\boldsymbol{u}})$ and $p(\underline{\boldsymbol{y}}|\underline{\boldsymbol{\phi}},\boldsymbol{\kappa},\underline{\boldsymbol{u}}^{*})$ that we denote as ρ_l.

After this step, we increase the number of particles such that the variance of the total log-likelihood is below a threshold. The typical threshold for the total log-likelihood is usually $\left(\sigma^{\text{opt}}\right)^2 = 2.16^2/(1-(\rho^l)^2)$[37,38]. To make this individualised we divide through by M, such that per individual the threshold is $\left(\sigma_m^{\text{opt}}\right)^2 = 2.16^2/\left(M\left(1-{\rho_m^l}^2\right)\right)$, which is justified by a normality assumption where $\left(\sigma^{\text{opt}}\right)^2 = \sum_{m=1}^{M}\left(\sigma_m^{\text{opt}}\right)^2$.

Initial state

A good initial guess helps sampling algorithms work more reliably and efficiently. As such, we estimate $\theta_{m,1}$ for each individual m using the logistic transformation of their initial value. For values lying on the boundaries (e.g., $k = a, b$ as defined previously) we perturb the value slightly so that it is within the boundaries such that a logistic function can be applied. Similarly, the initial value for $\theta_{m,3}$ for each individual was taken as the mean of their observations after performing a logistic transformation in a similar method to above. Constructing good parameter guesses for the other variables is difficult and thus we used a trial-and-error process until we had values that were reasonably close to the final results we were obtaining across multiple runs.

Testing of the sampling scheme

The sampling scheme was tested by recovering parameters for simulated data. The simulated data was designed to follow similar patterns to the observed suicidal ideation trajectories within Innowell. We outline the testing procedure and results for the random effects Wiener process model that was used as our final model, but similar testing was completed for the other models.

First, we assume that there are $M^{\mathrm{sim}} = 3000$ individuals. The number of observations for each individual was assumed to follow a negative binomial distribution with $T_m^{\mathrm{sim}} \sim NB(r = 0.7778, p = 0.1375)$, where r and p are the maximum likelihood estimated values using the suicidal ideation data. We then take all individuals with greater than 10 observations ($M^{\mathrm{sim}} = 49$). The time interval between observations is assumed to be constant with $\Delta t = 7$ days. The true population parameters were set to; $\eta^{\mathrm{sim}} = \{\{-2.5,1.0\},\{-1.0,1.0\}\}$. The parameters for each individual were drawn from the population distributions conditional on the population parameters. We then simulated trajectories in accordance with the trajectory dynamics and measurement processes defined above.

The sampling scheme was ran four times. Each run was performed for 5×10^4 iterations with 10% of chains removed as burn-in. We examined the convergence and resolution of these samples[39]. We used the Gelman-Rubin potential scale reduction factor ($\hat{R}_{\cdot}$) and it's multivariate equivalent ($\hat{R}_{\cdot}^{\mathrm{p}}$) as a quantitative measure of convergence[40,41]. These measures have values $\hat{R}_{\cdot}, \hat{R}_{\cdot}^{\mathrm{p}} > 1$ with convergence indicated by at least $\hat{R}_{\cdot}, \hat{R}_{\cdot}^{\mathrm{p}} < 1.1$. We calculated the effective sample size ($n_{\cdot}^{\mathrm{eff}}$) to determine that we have enough (typically $n_{\cdot}^{\mathrm{eff}} \gtrsim 1000$) samples to estimate the centre and 95% equal tailed credible intervals. We found reasonable convergence and resolution for the total log-likelihood ($\hat{R}_l$, 1.00001; n_l^{eff}, 12945), η ($\hat{R}_{\eta}^{\mathrm{p}}$, 1.0009; median$\left(\{n_{\eta,j}^{\mathrm{eff}} | j \in J_{\eta}\}\right)$, 13789), and the typical values across ϕ per individual (median$\left(\left\{\hat{R}_{\phi,m}^{\mathrm{p}} | m = 1, \dots, M\right\}\right)$, 1.0002; median$\left(\{n_{\phi,m,j}^{\mathrm{eff}} | m = 1, \dots, M; j \in J_{\eta}\}\right)$, 14099).

We then checked that the true values were consistent with the estimated posterior distribution. For the population parameters we found that all true values were within the 95% ETI. For the individual parameters we calculated the number of times that the true values were within the 50% ETI. Consistent with expectation we found that 50.1% (SE, 1.9%) of parameters met this criterion. Thus, the estimated posterior distribution has support over the true values.

Diagnostic tests of the sampling scheme for the Innowell data

The reported results for the Innowell data come from four separate sampling chains that were run for 10^5 iterations. Every iteration was saved for the log-likelihood and population parameters. As the number of individual parameters is large, we limited memory usage by only saving every 10th iteration for $\underline{\boldsymbol{\phi}}$. We removed the first 10% of the samples as a burn-in. We presupposed the percentage samples to remove as a burn-in but checked that this was

greater than the Raftery-Lewis criteria[42]. All estimated values are calculated after combining the remaining samples from each chain.

We examined the convergence and resolution of these samples using the same techniques as in the previous section[39]. We found reasonable convergence and resolution for the total log-likelihood ($\hat{R}_l$, 1.002; n_l^{eff}, 13455), η ($\hat{R}_\eta^{\mathrm{p}}$, 1.001; median$\left(\left\{n_{\eta,j}^{\mathrm{eff}} | j \in J_\eta\right\}\right)$, 12975), and the typical values across ϕ per individual (median$\left(\left\{\hat{R}_{\phi,m}^{\mathrm{p}} | m = 1, \ldots, M\right\}\right)$, 1.0002; median$\left(\left\{n_{\phi,m,j}^{\mathrm{eff}} | m = 1, \ldots, M; j \in J_\eta\right\}\right)$, 26946). We also qualitatively checked the trace plots for these values by eye. We perform similar checks for other quantities of interest that are reported throughout the paper, including for the predicted trajectories shown in Figure 2 and 3.

We also performed posterior predictive checks[43,44] to further understand the model specification. We checked whether a follow-up observation at the next time lies within the 95% ETI of the posterior predictive distribution given all prior observations for each observation where $t > 0$. We found that 96.1% (SD, 0.7%) met this criterion, which is consistent with the expected 95% value.

Supplementary Methods 4.

The aim of posterior prediction is to estimate the distribution for a future observation while incorporating information from the parameter posterior distribution. Specifically, sampling a future observation $\tilde{y}_{m,t}$ for individual m at time $t \geq T_m$ is given by the distribution,

$$p\left(\tilde{y}_{m,t}\middle|\underline{\boldsymbol{y}}\right) = \int g_{a,b}\left(\tilde{y}_{m,t}\middle|\tilde{x}_{m,t}\right) f\left(\tilde{x}_{m,t}\middle|\tilde{x}_{m,T_m}, q(\boldsymbol{\theta}_m|\phi_m), \boldsymbol{u}_{m,t}\right) \\ \times p\left(\boldsymbol{\phi}_m\middle|\underline{\boldsymbol{y}}\right) z_1\left(u_{m,t,i}\right) d\boldsymbol{\theta}_m d\tilde{x}_{m,t} dx_{m,T_m} du_{m,t} \tag{14}$$

where the posterior distribution for $\boldsymbol{\theta}_m$ is,

$$p\left(\boldsymbol{\phi}_m\middle|\underline{\boldsymbol{y}}\right) = \int p(\boldsymbol{\phi}_m|\boldsymbol{\eta}) p\left(\underline{\boldsymbol{\phi}}, \underline{\boldsymbol{\kappa}}, \underline{\boldsymbol{\eta}}\middle|\underline{\boldsymbol{y}}\right) d\boldsymbol{\kappa} d\boldsymbol{\eta}\,. \tag{15}$$

also $z_1\left(u_{m,t}\right) \sim N(u_{m,t}; 0, 1)$ is the distribution for a scalar particle rather than a vector of particles.

Computationally, this is completed by building the posterior distribution using the samples from the scheme described previously. Then, for each sample we use the model parameter values to simulate the predicted value. This yields a distribution of predicted values consistent with $p\left(\tilde{y}_{m,T}\middle|\underline{\boldsymbol{y}}\right)$. The summary statistics, such as the median or equal tailed intervals, are then estimated from that simulated distribution.

When we provide prediction estimates for an individual as their data is updated as in Figure 3 we make a further assumption. In that case, we set the population parameters to the MAP estimate. The above equation then simplifies to,

$$p\left(\tilde{y}_{m,t}\middle|\underline{\boldsymbol{y}}\right) \approx p\left(\tilde{y}_{m,t}\middle|\underline{\boldsymbol{y}}, \boldsymbol{\kappa}^{MAP}, \boldsymbol{\eta}^{MAP}\right) \tag{16}$$

which leads to the simplification of the posterior distribution for $\boldsymbol{\phi}_m$ as,

$$p\left(\boldsymbol{\phi}_m\middle|\underline{\boldsymbol{y}}\right) \approx p(\boldsymbol{\phi}_m|\boldsymbol{y}_m, \boldsymbol{\kappa}^{\mathrm{MAP}}, \boldsymbol{\eta}^{\mathrm{MAP}}) \tag{17}$$

where,

$$\boldsymbol{\eta}^{\mathrm{MAP}}, \boldsymbol{\kappa}^{\mathrm{MAP}} = \arg_{\boldsymbol{\eta},\boldsymbol{\kappa}} \max p\left(\underline{\boldsymbol{\phi}}, \boldsymbol{\kappa}, \boldsymbol{\eta}\middle|\underline{\boldsymbol{y}}\right). \tag{18}$$

Therefore, the uncertainties for κ and η are not passed through.

Supplementary Methods 5.

The marginalised distributions in Figure 1 in the main text show the predictive prior distribution for the j-th parameter for a hypothetical individual m that is outside the fitted population. This is given by,

$$p\left(\tilde{\phi}_{m,j}|\underline{\boldsymbol{y}}\right) = \int p(\tilde{\phi}_{m,j}|\mu_j,\tau_j)p\left(\mu_j,\tau_j\middle|\underline{\boldsymbol{y}}\right) d\mu_j d\tau_j \qquad (19)$$

which is simulated using our posterior samples for μ_j and τ_j.

Supplementary References

1. Capon, W. *et al.* Characterising variability in youth mental health service populations: A detailed and scalable approach using digital technology. *Australas. Psychiatry* 1–7 (2023) doi:10.1177/10398562231167681.
2. Wille, N. *et al.* Development of the EQ-5D-Y: A child-friendly version of the EQ-5D. *Qual. Life Res.* **19**, 875–886 (2010).
3. Van Spijker, B. A. J. *et al.* The Suicidal Ideation Attributes Scale (SIDAS): Community-based validation study of a new scale for the measurement of suicidal ideation. *Suicide Life-Threatening Behav.* **44**, 408–419 (2014).
4. Posner, K., Brown, G. K. & Stanley, B. The Columbia–Suicide Severity Rating Scale: Initial Validity and Internal Consistency Findings From Three Multisite Studies With Adolescents and Adults. *Am. J. Psychiatry* **168**, 1267–1277 (2011).
5. Kessler, R. C. *et al.* Short screening scales to monitor population prevalences and trends in non-specific psychological distress. *Psychol. Med.* **32**, 959–976 (2002).
6. Andrews, G. & Slade, T. Interpreting scores on the Kessler Psychological Distress Scale (K10). *Aust. N. Z. J. Public Health* **25**, 494–497 (2001).
7. Ising, H. K. *et al.* The validity of the 16-item version of the prodromal questionnaire (PQ-16) to screen for ultra high risk of developing psychosis in the general help-seeking population. *Schizophr. Bull.* **38**, 1288–1296 (2012).
8. Howie, C., Hanna, D., Shannon, C., Davidson, G. & Mulholland, C. The Structure of the Prodromal Questionnaire-16 (PQ-16): Exploratory and confirmatory factor analyses in a general non-help-seeking population sample. *Early Interv. Psychiatry* **16**, 239–246 (2022).
9. Altman, E. G., Hedeker, D., Peterson, J. L. & Davis, J. M. The altman self-rating Mania scale. *Biol. Psychiatry* **42**, 948–955 (1997).
10. OECD. Youth not in employment, education or training (NEET). (2023) doi:https://doi.org/10.1787/72d1033a-en.
11. Mundt, J. C., Marks, I. M., Shear, M. K. & Greist, J. H. The Work and Social Adjustment Scale: A simple measure of impairment in functioning. *Br. J. Psychiatry* **180**, 461–464 (2002).
12. Goldman, H. H., Skodol, A. E. & Lave, T. R. Revising axis V for DSM-IV: A review of measures of social functioning. *Am. J. Psychiatry* **149**, 1148–1156 (1992).
13. Humeniuk, R. *et al.* Validation of the alcohol, smoking and substance involvement screening test (ASSIST). *Addiction* **103**, 1039–1047 (2008).
14. Babor, T. F., Higgins-Biddle, J. C., Saunders, J. B. & Monteiro, M. G. *AUDIT: the Alcohol Use Disorders Identification Test: guidelines for use in primary health care*. *Guidelines for Use in Primary Care (second edition)* (2001).
15. Bush, K. *et al.* The AUDIT Alcohol Consumption Questions (AUDIT-C): An Effective Brief Screening Test for Problem Drinking. *Arch Intern Med.* **158**, 1789–1795 (1998).
16. Schuster, T. L., Kessler, R. C. & Aseltine, R. H. Supportive interactions, negative interactions, and depressed mood. *Am. J. Community Psychol.* **18**, 423–438 (1990).
17. Rush, A. J. *et al.* The 16-item Quick Inventory of Depressive Symptomatology (QIDS), clinician rating (QIDS-C), and self-report (QIDS-SR): A psychometric evaluation in patients with chronic major depression. *Biol. Psychiatry* **54**, 573–583 (2003).
18. Brown, E. S. *et al.* The Quick Inventory of Depressive Symptomatology-Self-report: A psychometric evaluation in patients with asthma and major depressive disorder. *Ann.*

Allergy, Asthma Immunol. **100**, 433–438 (2008).
19. Norman, S. B., Cissell, S. H., Means-Christensen, A. J. & Stein, M. B. Development and Validation of an Overall Anxiety Severity and Impairment Scale (OASIS). *Depress. Anxiety* **23**, 245–249 (2006).
20. Campbell-Sills, L. *et al.* Validation of a brief measure of anxiety-related severity and impairment: The Overall Anxiety Severity and Impairment Scale (OASIS). *J. Affect. Disord.* **112**, 92–101 (2009).
21. Body mass index (BMI) and waist measurement. https://www.health.gov.au/topics/overweight-and-obesity/bmi-and-waist.
22. Craig, C. L. *et al.* International physical activity questionnaire: 12-Country reliability and validity. *Med. Sci. Sports Exerc.* **35**, 1381–1395 (2003).
23. Buysse, D. J., Reynolds, C. F., Monk, T. H., Berman, S. R. & Kupfer, D. J. The Pittsburgh Sleep Quality Index: a new instrument for psychiatric practice and research. Psychiatry Res. 1989;28:193–213. *Psychiatry Res.* **28**, 193–213 (1989).
24. Roenneberg, T., Wirz-Justice, A. & Merrow, M. Life between clocks: Daily temporal patterns of human chronotypes. *J. Biol. Rhythms* **18**, 80–90 (2003).
25. Prins, A. *et al.* The Primary Care PTSD Screen for DSM-5 (PC-PTSD-5): Development and Evaluation Within a Veteran Primary Care Sample. *J. Gen. Intern. Med.* **31**, 1206–1211 (2016).
26. Hay, P. J., Mond, J., Buttner, P. & Darby, A. Eating disorder behaviors are increasing: Findings from two sequential community surveys in South Australia. *PLoS One* **3**, 1–5 (2008).
27. Arnold, L. *Stochastic Differential Equations: Theory and Applications*. *SIAM Review* (Wiley-Interscience, 1974). doi:10.1137/1018036.
28. Doucet, A. & Johansen, A. M. A Tutorial on Particle Filtering and Smoothing. in *Handbook of nonlinear filtering* 3 (2009).
29. Murray, L. M., Lee, A. & Jacob, P. E. Parallel Resampling in the Particle Filter. *J. Comput. Graph. Stat.* **25**, 789–805 (2016).
30. Andrieu, C., Doucet, A. & Holenstein, R. Particle Markov chain Monte Carlo methods. *J. R. Stat. Soc. Ser. B Stat. Methodol.* **72**, 269–342 (2010).
31. Michaud, N., de Valpine, P., Turek, D., Paciorek, C. J. & Nguyen, D. Sequential Monte Carlo Methods in the nimble and nimbleSMC R Packages. *J. Stat. Softw.* **100**, 1–32 (2021).
32. Chen, B., Dang, L., Zheng, N. & Principe, J. C. *Kalman Filtering Under Information Theoretic Criteria*. (Springer, Cham, 2023). doi:10.1007/978-3-031-33764-2_2.
33. Deligiannidis, G., Doucet, A. & Pitt, M. K. The correlated pseudomarginal method. *J. R. Stat. Soc. Ser. B Stat. Methodol.* **80**, 839–870 (2018).
34. Wiqvist, S., Golightly, A., McLean, A. T. & Picchini, U. Efficient inference for stochastic differential equation mixed-effects models using correlated particle pseudo-marginal algorithms. *Comput. Stat. Data Anal.* **157**, 107151 (2021).
35. Botha, I., Kohn, R. & Drovandi, C. Particle Methods for Stochastic Differential Equation Mixed Effects Models. *Bayesian Anal.* **16**, 575–609 (2021).
36. Doucet, A., Pitt, M. K., Deligiannidis, G. & Kohn, R. Efficient implementation of Markov chain Monte Carlo when using an unbiased likelihood estimator. *Biometrika* **102**, 295–313 (2015).
37. Tran, M.-N., Kohn, R., Quiroz, M. & Villani, M. The Block Pseudo-Marginal Sampler. (2016).

38. Choppala, P., Gunawan, D., Chen, J., Tran, M.-N. & Kohn, R. Bayesian Inference for State Space Models using Block and Correlated Pseudo Marginal Methods. 1–41 (2016).
39. Kruschke, J. K. Bayesian Analysis Reporting Guidelines. *Nat. Hum. Behav.* **5**, 1282–1291 (2021).
40. Gelman, A., Rubin, D. B., Gelman, A. & Rubin, D. B. Inference from Iterative Simulation Using Multiple Sequences Linked references are available on JSTOR for this article : Inference from Iterative Simulation Using Multiple Sequences. *Stat. Sci.* **7**, 457–472 (1992).
41. Brooks, S. P. & Gelman, A. General methods for monitoring convergence of iterative simulations. *J. Comput. Graph. Stat.* **7**, 434–455 (1998).
42. Raftery, A. E. & Lewis, S. M. The number of iterations, convergence diagnostics and generic Metropolis algorithms. *Pract. Markov Chain Monte Carlo* **7**, 763–773 (1995).
43. Box, G. E. P. Sampling and Bayes ' Inference in Scientific Modelling and Robustness. **143**, 383–430 (1980).
44. Gelman, A., Meng, X. L. & Stern, H. Posterior predictive assessment of model fitness via realized discrepancies. *Stat. Sin.* **6**, 733–807 (1996).
